# Does discovery of differentially culturable M tuberculosis really demand a new treatment paradigm? Longitudinal analysis of DNA clearance from sputum

**DOI:** 10.1186/s12879-018-3213-7

**Published:** 2018-07-03

**Authors:** Nicholas D. Walter, Camille M. Moore, Xavier A. Kayigire, Christian Dide-Agossou, William Worodria, Laurence Huang, Charles K. Everett, Gary S. Schoolnik, Payam Nahid, J. Lucian Davis

**Affiliations:** 1 Pulmonary Section, Denver VA Medical Center, Denver, CO USA; 2 Division of Pulmonary Sciences and Critical Care Medicine, University of Colorado Denver, Aurora, CO USA; 3 Division of Biostatistics and Bioinformatics, National Jewish Health, Denver, CO USA; 4 Department of Epidemiology, Colorado School of Public Health, Aurora, CO USA; 5 Infectious Disease Research Collaboration, Kampala, Uganda; 6 Department of Medicine, Mulago Hospital, Makerere University, Kampala, Uganda; 7 Division of Pulmonary and Critical Care Medicine, San Francisco General Hospital, University of California San Francisco, San Francisco, CA USA; 8 Pulmonary Associates, Burlingame, CA USA; 9 Department of Microbiology and Immunology, Stanford University School of Medicine, Stanford, California, USA; 10 Yale School of Public Health, New Haven, CT USA; 11 Division of Pulmonary, Critical Care, and Sleep Medicine Section, Yale School of Medicine, New Haven, CT USA; 12 HIV, Infectious Diseases, and Global Medicine Division, San Francisco General Hospital, University of California San Francisco, San Francisco, CA USA

**Keywords:** Tuberculosis, *Mycobacterium tuberculosis*, Treatment, Sputum, Drug tolerance, Differentially culturable

## Abstract

**Background:**

According to the traditional tuberculosis (TB) treatment paradigm, the initial doses of treatment rapidly kill most *Mycobacterium tuberculosis* (*Mtb*) bacilli in sputum, yet many more months of daily treatment are required to eliminate a small, residual subpopulation of drug-tolerant bacilli. This paradigm has recently been challenged following the discovery that up to 90% of *Mtb* bacilli in sputum are culturable only with growth-factor supplementation. These “differentially culturable” bacilli are hypothesized to be more drug-tolerant than routinely culturable bacilli. This hypothesis implies an alternative paradigm in which TB treatment does not rapidly reduce the total *Mtb* population but only the small, routinely culturable subpopulation. To evaluate these competing paradigms, we developed a culture-independent method for quantifying the viable fraction of *Mtb* bacilli in sputum during treatment.

**Methods:**

We used GeneXpert MTB/RIF to quantify *Mtb* DNA in sputa collected longitudinally from Ugandan adults taking standard 4-drug treatment for drug-susceptible pulmonary TB. We modeled GeneXpert cycle thresholds over time using nonlinear mixed-effects regression. We adjusted these models for clearance of DNA from killed-but-not-yet-degraded bacilli, assuming clearance half-lives ranging from 0 to 1.25 days. We used a convolution integral to quantify DNA from viable bacilli only, and converted cycle thresholds to *Mtb* genomic equivalents. We replicated our results in a South African cohort.

**Results:**

We enrolled 41 TB patients in Uganda. Assuming a DNA-clearance half-life of 0 days, genomic equivalents of viable sputum bacilli decreased by 0.22 log/day until 8.8 days, then by 0.07 log/day afterwards. Assuming a DNA-clearance half-life of 1.25 days, genomic equivalents of viable bacilli decreased by 0.36 log/day until 5.0 days, then by 0.06 log/day afterwards. By day 7, viable *Mtb* had decreased by 97.2–98.8%. We found similar results for 19 TB patients in South Africa.

**Discussion:**

Using a culture-independent method, we found that TB treatment rapidly eliminates most viable *Mtb* in sputum. These findings are incompatible with the hypothesis that differentially culturable bacilli are drug-tolerant*.*

**Conclusions:**

A culture-independent method for measuring viable *Mtb* in sputum during treatment corroborates the traditional TB treatment paradigm in which a rapid bactericidal phase precedes slow, elimination of a small, residual bacillary subpopulation.

**Electronic supplementary material:**

The online version of this article (10.1186/s12879-018-3213-7) contains supplementary material, which is available to authorized users.

## Background

Traditional understanding of tuberculosis (TB) treatment has recently been challenged by reports that up to 90% of *Mtb* in the sputum of treatment-naïve patients may be non-culturable on standard agar plates, growing only with growth factor supplementation (“differentially culturable”) [1–5]. The traditional TB treatment paradigm is based on enumeration of *Mycobacterium tuberculosis* (*Mtb*) that grows on agar plates (routinely culturable *Mtb*). This traditional measure of sputum bacillary load suggests that killing is biphasic. The initial 5–7 day bactericidal phase appears to kill ~ 99% of *Mtb* in sputum [6–8]. During the subsequent, “sterilizing” phase of treatment, the rate of killing appears to slow by at least 80% [9]. The residual population is comprised of drug-tolerant “persister” phenotypes that are capable of surviving prolonged antibiotic exposure despite an absence of drug-resistance mutations [10, 11]. Critically, the traditional biphasic killing paradigm hinges on the assumption that enumeration of routinely culturable *Mtb* accurately represents killing in the total *Mtb* population of sputum (i.e.*,* it does not account for an additional, differentially culturable, component of sputum).

The discovery of differentially culturable *Mtb* has raised concern that the traditional paradigm may be based on an incomplete and potentially misleading measure of *Mtb* burden. The differentially culturable *Mtb* population is particularly important if it responds to treatment differently than routinely culturable *Mtb*. Recent in vitro evidence suggests differentially culturable *Mtb* may be more drug tolerant than routinely culturable *Mtb* [3, 12]. This hypothesis implies an alternative paradigm in which drug-tolerant *Mtb* phenotypes dominate sputum even prior to TB treatment [1]. This paradigm suggests that treatment does not rapidly decrease the total burden of *Mtb* in sputum; only the small routinely culturable fraction is rapidly and selectively eliminated [13]. Validation of this alternative paradigm would fundamentally reshape our understanding of TB treatment and have critical implications for drug development [13].

A key question is whether the hypothesized drug tolerance of differentially culturable *Mtb* has practical significance in patients with TB. Specifically, are differentially culturable *Mtb* phenotypes sufficiently drug tolerant in vivo that the traditional biphasic killing paradigm should be questioned? For the traditional paradigm to remain valid, routinely culturable and differentially culturable *Mtb* would have to be killed at roughly similar rates. By contrast, if differentially culturable *Mtb* are killed much more slowly, enumeration of only routinely culturable *Mtb* would misrepresent change in the total *Mtb* burden of sputum. This would favor the alternative paradigm that the total *Mtb* burden of sputum does not decrease rapidly.

To re-evaluate these paradigms in a culture-independent manner, we developed a new approach to characterizing the rates and phases of *Mtb* killing during treatment for active TB based on *Mtb* DNA abundance in sputum. Quantification of *Mtb* DNA enumerates the total *Mtb* population (irrespective of whether bacilli are routinely culturable or differentially culturable). Using a conceptually simple mathematical model, we adjust our estimates of the quantity of *Mtb* for the characteristic of DNA that is often cited as limiting its usefulness as a measure of treatment response – its slow degradation after cell death [14]. Our analysis does not address what proportion of *Mtb* in sputum is differentially culturable; instead we sought evidence that the differentially culturable *Mtb* population is cleared from sputum substantially more slowly than routinely culturable *Mtb.* The resulting culture-independent estimates of early killing support the traditional biphasic killing paradigm [6–8].

## Methods

### Study design and enrollment

Our primary analysis enrolled consecutive adults hospitalized with drug-susceptible pulmonary TB at Mulago National Referral Hospital in Kampala, Uganda as previously described [15]. Patients provided sputa and a NALC-NaOH processed pellet was treated with sample reagent and tested with GeneXpert MTB/RIF (Version G4, Cepheid, Sunnyvale, CA USA) testing before treatment (day 0) and after 2, 4, 7, 14, 28 and 56 daily standard doses of isoniazid, rifampicin, pyrazinamide, and ethambutol. To determine whether our results were reproducible, we selected a previously-published study in South Africa as a replication cohort. The South African study evaluated the effect of pre-treatment with propidium monoazide (PMA) on GeneXpert MTB/RIF (also Version G4) [16]. Our analysis used only data from 19 patients with non-PMA treated (control) samples obtained at day 0 and after 3, 7, 14, 28, 35, and 56 days of treatment.

### Conceptual basis for using DNA to estimate viable Mtb

Before treatment, the burden of *Mtb* DNA in sputum predominantly reflects the burden of viable bacilli. During treatment, measured DNA has two components: DNA from viable *Mtb* and residual DNA from dead *Mtb*. We aimed to estimate the viable and dead components. We defined the half-life of DNA clearance as days required for a 50% reduction in DNA from dead *Mtb*, due to either expectoration or degradation by active host processes. For any DNA burden at any time point, the longer the DNA clearance half-life is, the larger the dead component and the smaller the viable component must be. Using this construct and assuming a range of plausible clearance rates, we estimated the burden of viable *Mtb* over time.

### Analysis

We modeled mean cycle-threshold (CT) values for GeneXpert during the first 56 days of treatment using a nonlinear mixed-effects model framework. Conceptually, mean cycle threshold is inversely proportional to the logarithm of the number of DNA copies. Therefore we modeled CT values as a linear function of the logarithm of the total DNA (viable + dead) at time *t* (See Additional file 1 for details). We assumed that the viable component of DNA in sputum decreased exponentially with treatment, and allowed the rate of decrease in DNA to change from a higher to a lower rate at a single time point; this change point was not pre-specified, but rather was estimated as a parameter in the model. We used a convolution integral to solve for the component of non-degraded DNA from dead bacteria at a given time *t*. We evaluated five possible DNA clearance half-lives: zero, 0.5, 0.75, 1, and 1.25 days. A clearance half-life of zero days represents the extreme and unrealistic assumption that DNA from dead *Mtb* is degraded instantaneously, implying that all *Mtb* DNA originates from viable bacilli. We used a likelihood-based approach assuming a Gaussian distribution to account for right censoring of Xpert CT values at 41 cycles. In addition, our models included a random intercept for each subject to account for correlation due to repeated measures made on subjects over time. Our base models assumed that, prior to treatment, 99% of *Mtb* DNA in sputum arose from living bacteria*.* In sensitivity analyses, we fit alternative models in which 80% or 90% of *Mtb* in treatment-naïve sputum was assumed to be viable*.* We fit all models in SAS 9.3 Proc NLMIXED.

### Conversion of GeneXpert MTB/RIF to genomic equivalents

For the purposes of conceptual illustration only, we converted estimated CT values from our models to a measure of bacillary burden (“genomic equivalents”) based on the data previously presented by Blakemore et al. showing a linear relationship between log_10_ *Mtb* and Xpert CT values [17].

## Results

For our primary analysis, 41 Ugandan adults with drug-susceptible, culture-positive pulmonary TB were enrolled. Twenty-three (56%) were persons living with HIV. The proportion with a positive sputum Xpert result declined gradually, from 100% at baseline to 80% at four weeks to 50% at eight weeks. The previously-published replication cohort included 19 South African patients with drug-susceptible TB and serial Xpert testing performed without PMA pre-treatment [16].

**Fig. 1:**
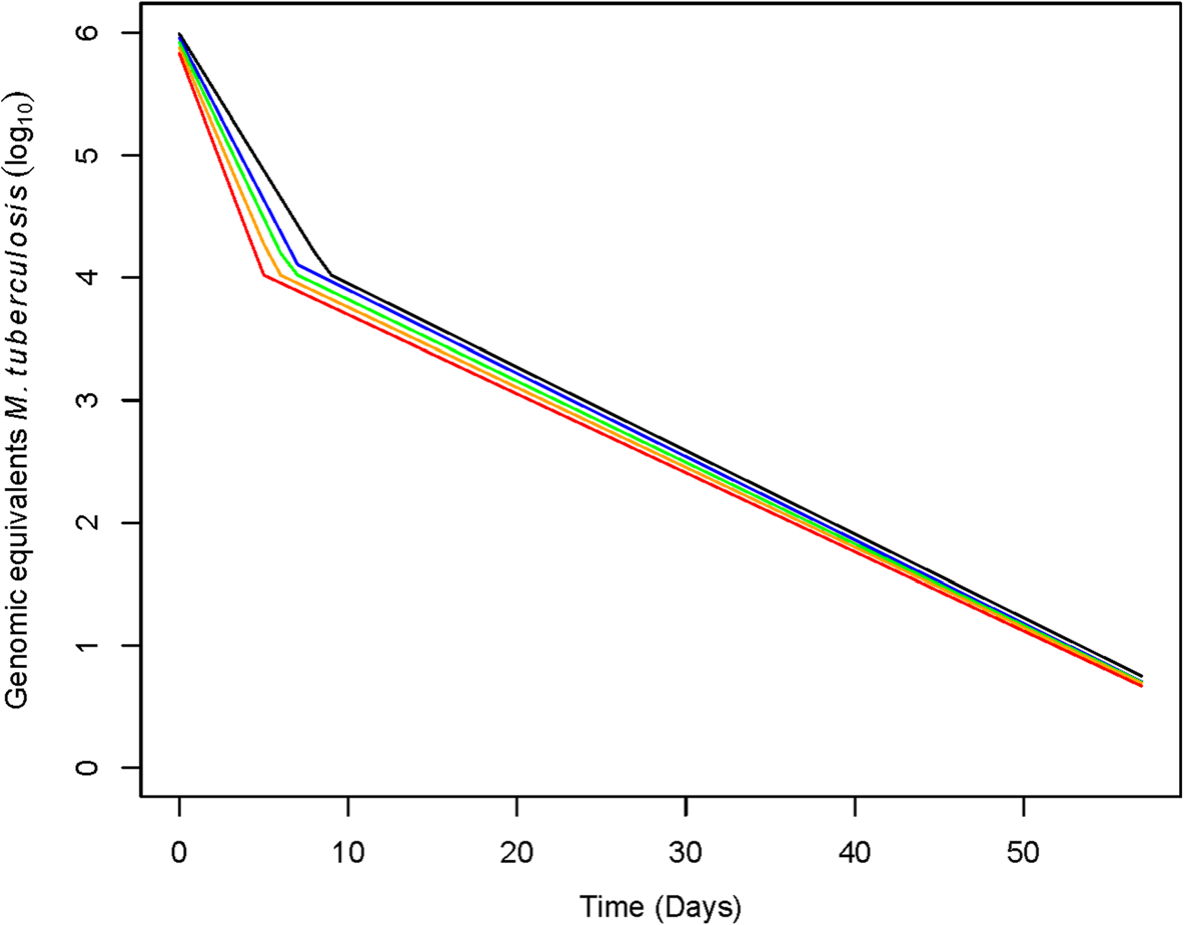
Estimated viable *Mtb* in sputum during the first 56 days of treatment for drug-susceptible TB among 41 Ugandan adults based on GeneXpert data. Models assumed different rates of clearance of DNA from dead *Mtb* ranging from instantaneous (black) to 0.5 days (blue) to 0.75 days (green), 1 day (orange), to 1.25 days (red)

With the half-life of DNA clearance assumed to be zero, the viable component of *Mtb* DNA in sputum initially decreased rapidly (− 0.22 log/day) among Ugandan patients. There was a change point at 8.8 days, after which the rate of decline slowed to − 0.07 log/day (Fig. 1 & Table 1). Even in this extreme case that assumed no dead component*,* viable *Mtb* decreased 97.2% from baseline to day 7. With more plausible estimates for the half-life of *Mtb* DNA clearance, the initial rate of killing was faster and the change point occurred earlier. For example, with a DNA clearance half-life of 1.25 days, the initial rate of killing was − 0.36 log/day) and the change point was 5.0 days. When the half-life was extended to 1.5 days, the model estimated no viable component at 7 days, inconsistent with the positive culture results obtained from each individual. The rate of killing was 69.4–82.2% slower in the later killing period relative to the early killing period. With each increase in the half-life of DNA clearance, the differences between the rates in the early and late periods increased.

**Table 1:** Estimated burden of viable and dead *Mtb* and rates of killing, assuming different rates of DNA clearance among Ugandan patients with drug-susceptible TB

DNA clearance rate (days)	Genome equivalent viable *Mtb* at day 7 ^a^ (95% CI)	Genome equivalent dead *Mtb* at day 7 ^b^ (95% CI)	Reduction in viable *Mtb* by day 7		Change point in days^c^ (95% CI)	Early rate of killing ^d^(log_10_/day)	Late rate of killing ^e^(log_10_/day)	% decrease in rate of killing ^f^	p-val for difference in rates
			log_10_	%					
0	4.4 (3.6, 5.2)	0	−1.6 (− 2.3, − 0.9)	97.2% (85.8, 99.5)	8.8 (3.3, 14.2)	−0.22 (− 0.32, − 0.12)	−0.07 (− 0.09, − 0.05)	69.4%	0.004
0.5	4.1 (3.2, 4.9)	3.9 (2.2, 5.6)	− 1.9 (− 2.7, − 1.0)	98.6% (90.2, 99.8)	7 (2.7, 11.3)	−0.26 (− 0.38, − 0.14)	−0.07 (− 0.09, − 0.05)	74.2%	0.002
0.75	4.02 (3.2, 4.8)	4.1 (3.2, 4.9)	−1.9 (− 2.6, − 1.3)	98.7% (94.3, 99.7)	6.5 (3.4, 9.6)	− 0.29 (− 0.42, − 0.15)	−0.07 (− 0.08, − 0.05)	76.8%	0.003
1	4.0 (3.1, 4.8)	4.2 (3.4, 5.0)	−1.9 (− 2.6, − 1.3)	98.8% (94.6, 99.7)	5.7 (1.9, 9.6)	− 0.32 (− 0.53, − 0.12)	−0.07 (− 0.08, − 0.05)	79.6%	0.02
1.25	3.9 (3.1, 4.7)	4.4 (3.6, 5.1)	− 1.9 (− 2.6, − 1.3)	98.8% (94.7, 99.7)	5.0 (0.5, 9.5)	− 0.36 (− 0.67, − 0.05)	−0.06 (− 0.08, − 0.05)	82.2%	0.06

^a^Genome equivalents *Mtb* DNA remaining at day 7 expected to be from viable *Mtb* (log_10_)

^b^Genome equivalents *Mtb* DNA remaining at day 7 expected to be from dead *Mtb* (log_10_)

^c^Time point (in days) of change between early and late killing based on bi-exponential mixed effects models

^d^Rate of decline in genomic equivalents *Mtb* before change point

^e^Rate of decline in genomic equivalents *Mtb* after change point

^f^Late rate of killing relative to early rate of killing

In our replication cohort (19 South African patients), we also identified a biphasic pattern. With a clearance half-life of zero, the viable component decreased 92.1% (95% CI: 65.1–98.2%) by day 7. There was a significant change point at 17.3 days by which time the viable component had decreased 99.8% (95% CI: 93.8–100%). The number of observations was insufficient to fit models estimating the components of living bacilli and dead bacilli using half-life parameters.

## Discussion

Our analysis identified a sharp decline in the burden of viable Mtb DNA during the first days of standard TB treatment. This finding is incompatible with the hypothesis that pre-treatment sputum is dominated by drug-tolerant phenotypes and that only a small proportion of *Mtb* is killed rapidly during initial TB treatment. Since the total *Mtb* burden is rapidly reduced, we conclude that the differentially culturable fraction could not have a clinically-significant degree of drug tolerance. Put simply, a rapid decrease in *Mtb* DNA in sputum must indicate rapid killing, regardless of whether the bacteria routinely or differentially culturable.

Our analysis did not estimate what proportion of *Mtb* is differentially culturable. Rather we asked whether there is evidence in humans that differentially culturable *Mtb* is drug-tolerant to a degree that threatens the validity of the traditional culture-based biphasic paradigm of killing. The rapid clearance of total *Mtb* we observed suggests that the population of *Mtb* present at baseline could not be dominated by highly tolerant phenotypes. Our results do not imply that drug-tolerant phenotypes are absent from pre-treatment sputum. Instead, our analysis is consistent with the prevailing hypothesis that only a small fraction of *Mtb* in sputum is in a drug-tolerant phenotype at baseline.

The rapid initial decline in DNA we observe is consistent with declines observed with other culture-independent measures of bacterial burden, namely messenger RNA (mRNA) and ribosomal RNA (rRNA). For example, both our previous study [15] and another [18] found that *Mtb* mRNA abundance in sputum decreases > 99% by the fourth day of treatment. Among South African patients, Honeyborne et al. described an 88% reduction in *Mtb* 16S rRNA during the first three days of treatment [1]. Although slow-growing, drug-tolerant phenotypes likely have lower mRNA or rRNA content per viable bacillus than rapidly-killed phenotypes [19], the consistency of these changes in mRNA, rRNA and DNA with the changes observed in historical, culture-based studies reinforces the current finding that the differentially culturable fraction of sputum must not be highly drug-tolerant at baseline*.*

Our analysis has several limitations. First, there is uncertainty around our assumption that 99% of DNA is from viable *Mtb* prior to treatment. In sensitivity analyses described in Additional file 2, we tested other assumptions (e.g., prior to treatment 80% or 90% of DNA is from viable *Mtb*); these assumptions had minimal impact on our conclusions. Second, the actual half-life of DNA clearance is unknown and may also vary from patient to patient. Our models therefore evaluated a spectrum of possible half-lives, ranging from zero days (i.e., instantaneous degradation) to 1.25 days. Since we observed an average decline of 1.56 log DNA by day 7, the hypothetical upper bound of average DNA clearance half-life is 1.4 day (calculated by assuming that all *Mtb* is dead at baseline). Our DNA clearance half-life assumptions are compatible with the observation that patients may remain sputum Xpert positive well after treatment completion since many DNA clearance half-lives pass before Xpert MTB/RIF turns negative [14]. Finally, Xpert MTB/RIF CT values are not validated for quantification [20]. We therefore confirmed Xpert MTB/RIF results by measuring DNA abundance and rate of decline in longitudinal specimens in a subset (*n* = 17) of these patients using a validated qRT-PCR assay [15]. Models fit to qRT-PCR data recapitulated results based on Xpert MTB/RIF.

Our study also had several strengths. First, our approach enabled us to make culture-independent estimates of change in viable *Mtb* during treatment. The advantage is that this analysis is agnostic to the question of differential versus routine culturability. Recent identification of differentially culturable *Mtb* [1–5] has provided insights into heterogeneous *Mtb* populations but the implications of differentially culturable *Mtb* for patients and treatment paradigms has remained unclear. Our analysis suggests that – in patients – differentially culturable *Mtb* are unlikely to be killed much more slowly than routinely culturable *Mtb.* Second, we evaluated these insights in a second and distinct study population from South Africa collected by a different group of investigators, which increases the generalizability of our findings. Finally, we used rigorous methods to maximize the accuracy of quantification and frequently repeated sputum collection.

## Conclusions

Our culture-independent analysis supports the traditional paradigm that TB treatment involves a short bactericidal phase in which most *Mtb* bacilli are rapidly killed followed a prolonged sterilizing phase in which minority subpopulations of drug-tolerant phenotypes are slowly eliminated. Our results are not consistent with the recently-proposed alternative hypothesis, namely that most of the *Mtb* population of sputum from treatment-naïve patients is tolerant of and refractory to antimicrobial treatment at baseline. Routine culture on agar plates may fail to enumerate a significant fraction of the bacterial population, but the differentially culturable *Mtb* population does not appear to be highly drug-tolerant. Our analysis corroborates the longstanding conceptual model of drug-dependent *Mtb* killing. Confirming the correct model of killing is critically important to the global community’ efforts to expand the pipeline of new drug regimens for *Mtb.*

## Additional files

Additional file 1: Technical appendix. A methods supplement providing technical details of the model. (PDF 197 kb)

Additional file 2: **Table S1.** This is a supplemental table providing model outputs based on alternative assumptions that 80% or 90% of *Mtb* DNA in sputum prior to treatment initiation originates from viable bacilli. (DOCX 16 kb)

## Abbreviations

CT: Cycle threshold
mRNA: messenger RNA
*Mtb*: *Mycobacterium tuberculosis*
PMA: Propidium monoazide
rRNA: ribosomal RNA
TB: Tuberculosis
