# Supplementary material for: Does discovery of differentially culturable M tuberculosis really demand a new treatment paradigm? Longitudinal analysis of DNA clearance from sputum
Source: BMC Infect Dis. 2018 Jul 3;18:293. doi: 10.1186/s12879-018-3213-7 (PMC6029172; doi:10.1186/s12879-018-3213-7)
Supplement: Supplementary file 1 — Technical appendix. A methods supplement providing technical details of the model. (PDF 197 kb) [file 12879_2018_3213_MOESM1_ESM.pdf]

Additional File 1

Does discovery of differentially culturable *M. tuberculosis* demand a new treatment paradigm? Longitudinal analysis of DNA clearance from sputum (technical appendix)

Nicholas D. Walter, Camille M. Moore, Xavier A. Kayigire, Christian Dide Agossou, William Worodria, Laurence Huang, Charles K. Everett, Gary S. Schoolnik, Payam Nahid, J. Lucian Davis

Supplement: Models to estimate the viable and dead components of *M. tuberculosis* DNA in sputum.

Let $\mathcal{T}(t)$ be the total amount of *Mtb* DNA in a sputum sample taken at time t. Let $\mathcal{L}(t)$ and $\mathcal{D}(t)$ be the number of DNA counts from viable (live) and non-viable (dead) bacteria, respectively. Clearly, $\mathcal{T}(t) = \mathcal{L}(t) + \mathcal{D}(t)$.

Let p_0 be the proportion of DNA from viable bacteria at baseline, such that $L_0 = p_0 T_0$, where L_0 is the amount of DNA from viable bacteria at baseline and T_0 is the total amount DNA at baseline. Then $D_0 = (1-p_0)T_0$, where D_0 is the amount of DNA from non-viable bacteria at baseline. In order to fit our models, we assumed $p_0 = 0.99$ and performed sensitivity analyses assuming $p_0 = 0.8$ and 0.9.

DNA from Viable Bacteria

We assume that the number of bacteria decay exponentially, in a biphasic manner, such that *Mtb* are initially eliminated rapidly, during the bactericidal phase of antibiotic treatment, and later more slowly, during the the sterilizing phase of treatment. We assume that the shift between these two phases occurs at a change point, c. Then the amount of DNA from viable *Mtb* at time t is:

$$\mathcal{L}(t) = T_0 L(t)$$

where

$$L(t) = \begin{cases} p_0 e^{\alpha t} & \text{for } t \leq c \\ p_0 e^{\alpha t + \psi(t-c)} & \text{for } t > c \end{cases}$$

α is the rate of decay during the bactericidal phase and $\alpha + \psi$ is the rate of decay during the sterilizing phase.

DNA from Non-Viable Bacteria

The amount of DNA from non-viable *Mtb* at a given time depends on the decay or elimination of DNA from both non-viable bacteria present at baseline, as well as the continuous supply of DNA from newly killed *Mtb* due to the antibiotic treatment. We assume the rate of decay of DNA from non-viable *Mtb* is constant, following an exponential decay, with a rate parameter γ. We fit models assuming γ = -1.39, -0.92, -0.69, and -0.55, which correspond to half-lives of 0.5, 0.75, 1, and 1.25 days, respectively. Let

$$\mathcal{D}(t) = T_0 D(t)$$

where

$$D(t) = (1 - p_0)e^{\gamma t} + \int_0^t \left[-\frac{d}{d\tau} L(\tau) \right] e^{\gamma(t-\tau)} d\tau$$

The first term in the equation represents the decay of DNA from *Mtb* that were not viable at baseline and the convolution integral represents the DNA from non-viable *Mtb* that are present at time t due to killing of viable bacteria by the antibiotic treatment. Solving the integral:

$$D(t) = \begin{cases} (1-p_0)e^{\gamma t} - \frac{p_0 \alpha}{\alpha - \gamma}\left(e^{\alpha t} - e^{\gamma t}\right) & \text{for } t \leq c \\ (1-p_0)e^{\gamma t} - \frac{p_0 \alpha}{\alpha - \gamma}\left(e^{(\alpha-\gamma)c} - 1\right) e^{\gamma t} & \\ \quad - \frac{p_0(\alpha+\psi)}{\alpha+\psi-\gamma}\left(e^{(\alpha+\psi)(t-c)} - e^{\gamma(t-c)}\right) e^{\alpha c} & \text{for } t > c \end{cases}$$

.

Model to Account for Presence of DNA from Non-Viable Bacteria

Xpert measures the amount of *Mtb* DNA in a sputum sample from subject i at time j in terms of the number of PCR cycles, $Y(t_{ij})$, needed to reach the detection threshold, K. Therefore, we model $Y(t_{ij})$ as follows:

$$
\begin{aligned}
Y(t_{ij}) &= -\log_{0.5}(K) + \log_{0.5}(\mathcal{T}(t_{ij})) + \epsilon_{ij} \\
&= -\log_{0.5}(K) + \log_{0.5}(T_0) + u_i + \log_{0.5}\left(L(t_{ij}) + D(t_{ij})\right) + \epsilon_{ij} \\
&= \beta_0 + u_i + \log_{0.5}\left(L(t_{ij}) + D(t_{ij})\right) + \epsilon_{ij}
\end{aligned}
$$

where ϵ_{ij} is an independent, normally distributed error term. As we have longitudinal data on the subjects in our study, we account for correlation of repeated measurements by including a normally distributed, subject specific random intercept, u_i, in the model. This allows each subject to have their own specific baseline level of *Mtb* DNA.

We use a likelihood based approach based on the normal distribution to account for right censoring of $Y(t)$ at the limit of detection. All models were fit in SAS 9.3 Proc NLMixed.

Model Not Accounting for Presence of DNA from Non-Viable Bacteria

To assume that all DNA present is from live bacteria, let $p_0 = 1$ and take $\lim_{\gamma \to -\infty} Y(t_{ij})$. Then:

$$
Y(t_{ij}) = \beta_0 + u_i + \log_{0.5}\left(L(t_{ij})\right) + \epsilon_{ij}
$$
