# Supplementary material for: Does discovery of differentially culturable M tuberculosis really demand a new treatment paradigm? Longitudinal analysis of DNA clearance from sputum
Source: BMC Infect Dis. 2018 Jul 3;18:293. doi: 10.1186/s12879-018-3213-7 (PMC6029172; doi:10.1186/s12879-018-3213-7)
Supplement: Supplementary file 2 — Table S1. This is a supplemental table providing model outputs based on alternative assumptions that 80% or 90% of Mtb DNA in sputum prior to treatment initiation originates from viable bacilli. (DOCX 16 kb) [file 12879_2018_3213_MOESM2_ESM.docx]

Additional file 2

**Does discovery of differentially culturable M tuberculosis really demand a new treatment paradigm?**

**Longitudinal analysis of DNA clearance from sputum**

Nicholas D Walter, Camille Moore, Xavier A. Kayigire, Christian Dide Agossou, William Worodria, Laurence Huang, Charles K Everett, Gary S Schoolnik, Payam Nahid, J Lucian Davis

Our primary analysis assumed that, prior to treatment, 99% of *Mtb* DNA in sputum originates from viable rather than dead *Mtb.*  We conducted sensitivity analyses to test the influence of this assumption on our results. This supplemental table displays model outputs based on the alternative assumption that 80% or 90% of DNA in sputum originates from viable *Mtb.* We conclude that the percentage of *Mtb* DNA assumed to viable at baseline does not meaningfully impact our conclusions.

| **Supplemental Table**. Results of sensitivity analysis assuming that 80% or 90% of *Mtb* DNA in sputum prior to treatment initiation originates from viable bacilli. Estimated burden of viable and dead *Mtb* and rates of killing, assuming different rates of DNA clearance among Ugandan patients with drug-susceptible TB. | | | | | | | | | | |
| --- | --- | --- | --- | --- | --- | --- | --- | --- | --- | --- |
| % of *Mtb* DNA originating from viable bacilli at baseline | DNA clearance rate  (days) | Genome equivalent viable *Mtb* at day 7 ^a^ (95% CI) | Genome equivalent dead *Mtb* at day 7 ^b^ (95% CI) | Reduction in viable *Mtb* by day 7 | | Change point in days^c^  (95% CI) | Early rate of killing ^d^ (log_10_/day) | Late rate of killing ^e^ (log_10_/day) | % decrease in rate of killing ^f^ | p-val for difference in rates |
|  |  |  |  | log_10_ | % |  |  |  |  |  |
| 80% | 0.5 | 4.14  (3.25, 5.02) | 3.92  (2.00, 5.83) | -1.75  (-2.58, -0.92) | 98.2  (87.9, 99.7) | 7.2  (2.5, 11.8) | -0.25  (-0.37, -0.13) | -0.07  (-0.09, -0.05) | 72.8 | 0.004 |
| 80% | 0.75 | 4.03  (3.22, 4.83) | 4.04  (3.15, 4.93) | -1.82  (-2.47, -1.17) | 98.5  (93.2, 99.7) | 6.6  (3.5, 9.7) | -0.27  (-0.4, -0.14) | -0.07  (-0.08, -0.05) | 75.5 | 0.0038 |
| 80% | 1 | 3.96  (3.15, 4.77) | 4.21  (3.42, 4.99) | -1.84  (-2.49, -1.19) | 98.6  (93.6, 99.7) | 5.8  (1.9, 9.8) | -0.3  (-0.5, -0.11) | -0.07  (-0.08, -0.05) | 78.3 | 0.0206 |
| 80% | 1.25 | 3.89  (3.07, 4.72) | 4.36  (3.63, 5.1) | -1.85  (-2.51, -1.2) | 98.6  (93.6, 99.7) | 5.1  (0.4, 9.8) | -0.34  (-0.63, -0.05) | -0.06  (-0.08, -0.05) | 81.0 | 0.0673 |
| 90% | 0.5 | 4.12  (3.24, 5) | 3.89  (2.1, 5.68) | -1.8  (-2.64, -0.97) | 98.4  (89.3, 99.8) | 7.1  (2.7, 11.5) | -0.26  (-0.38, -0.14) | -0.07  (-0.09, -0.05) | 73.6 | 0.0029 |
| 90% | 0.75 | 4.02  (3.22, 4.83) | 4.05  (3.16, 4.93) | -1.86  (-2.51, -1.21) | 98.6  (93.9, 99.7) | 6.5  (3.4, 9.7) | -0.28  (-0.41, -0.15) | -0.07  (-0.08, -0.05) | 76.2 | 0.0033 |
| 90% | 1 | 3.96  (3.14, 4.77) | 4.21  (3.43, 5) | -1.89  (-2.54, -1.24) | 98.7  (94.2, 99.7) | 5.8  (1.9, 9.7) | -0.31  (-0.51, -0.11) | -0.07  (-0.08, -0.05) | 79.0 | 0.0191 |
| 90% | 1.25 | 3.89  (3.07, 4.72) | 4.37  (3.63, 5.1) | -1.9  (-2.56, -1.24) | 98.7  (94.2, 99.7) | 5.0  (0.5, 9.6) | -0.35  (-0.65, -0.05) | -0.06  (-0.08, -0.05) | 81.7 | 0.0633 |
